# Recent updates of centromere proteins in hepatocellular carcinoma: a review

**DOI:** 10.1186/s13027-024-00630-2

**Published:** 2025-02-06

**Authors:** Zhongyuan Yang, Wenjiao Chen, Yunhui Liu, Yuxin Niu

**Affiliations:** 1 Department of Infectious Diseases, Tongji Hospital, Tongji Medical College and State Key Laboratory for Diagnosis and Treatment of Severe Zoonostic Infectious Disease, Huazhong University of Science and Technology, 1095, Jiefang Avenue, Wuhan, 430030 Hubei China; 2 Department of Dermatology, Wuhan Hankou Hospital, Wuhan, Hubei China

**Keywords:** Hepatocellular carcinoma, Centromere protein, Chromosomal instability, Therapeutic target

## Abstract

Hepatocellular carcinoma (HCC) is the fourth leading cause of cancer-related death worldwide, with approximately 800,000 deaths worldwide each year. Owing to the atypical early symptoms and characteristics of HCC, over 80% of HCC patients cannot receive curative treatment. The treatment of HCC is facing a bottleneck, and new treatment methods are urgently needed. Since the pathogenesis of HCC is not yet clear, identifying the molecular mechanisms and therapeutic targets related to it is crucial. Centromeres are considered special deoxyribonucleic acid (DNA) sequences with highly repetitive sequences that are physically connected to the spindle during cell division, ensuring equal division of genetic material between daughter cells. The numerous proteins that aggregate on this sequence during cell division are called centromere proteins (CENPs). Currently, numerous studies have shown that CENPs are abnormally expressed in tumor cells and are associated with patient prognosis. The abnormal expression of CENPs is a key cause of chromosomal instability. Furthermore, chromosomal instability is a common characteristic of the majority of tumors. Chromosomal instability can lead to uncontrolled and sustained division and proliferation of malignant tumors. Therapeutic plans targeting CENPs play important roles in the treatment of HCC. For example, small ribonucleic acid (RNA) can silence CENP expression and prevent the occurrence and development of liver cancer. In recent years, studies of HCC-targeting CENPs have gradually increased but are still relatively novel, requiring further systematic elaboration. In this review, we provide a detailed introduction to the characteristics of CENPs and discuss their roles in HCC. In addition, we discuss their application prospects in future clinical practice.

## Introduction

The global cancer data for 2022 released by the International Agency for Research on Cancer show that among 185 countries and regions in the world, hepatocellular carcinoma (HCC) ranks sixth in incidence among all cancers, and HCC is one of the top three causes of cancer-related deaths [1]. At present, the overall annual incidence and mortality rates of HCC are both approximately 7% [2]. In the past decade, there has been a slow upward trend in Europe, Latin America, South Asia, and East Asia. The treatment methods for HCC include surgery, interventional therapy, targeted drugs, and immune checkpoint therapy (ICH). Early HCC can be treated by combining one or more of the above methods according to the patients’ condition. For example, early HCC can achieve a 5-year survival rate of over 70% through ablation or local resection. However, in advanced HCC, only approximately 30% of HCC patients have an objective response to treatment, and the 3-year overall survival rate is far below 50% with ICH therapy, indicating that there is still room for continuous improvement. Drug resistance in HCC treatment is one of the reasons for the poor prognosis of HCC patients, and its mechanisms include autocrine and paracrine LIF signaling, epigenetic regulation of TFR2, regulatory SLC7A11 transcription, and six2 overexpression [3–5]. Therefore, exploring the molecular mechanisms underlying the occurrence and development of HCC, as well as therapeutic targets, is crucial for the early detection and treatment of HCC. Centromeres are critical chromosomal sites in eukaryotes, where kinetochores form and attach to spindle microtubules to coordinate chromosome separation during mitosis and meiosis [6, 7]. Centromere proteins (CENPs) are a collective term containing many proteins assembled on centromere DNA. There are many members of the CENP family, such as CENP-A, CENP-E, CENP-F, CENP-L and CENP-U [8–13]. Currently, numerous studies have shown that abnormal expression of CENPs is one of the important mechanisms for chromosomal instability [14, 15]. When CENPs are abnormally expressed in the cell cycle, they may affect the cell cycle process and the normal separation of sister chromatids, thus leading to chromosomal instability, which is an important factor in tumorigenesis and neoplastic development [16–19]. Additionally, further research on CENPs in HCC patients confirms this viewpoint [20, 21]. In HCC, targeting CENPs has shown positive intervention effects. A study from China revealed that the CENP-E inhibitor GSK923295 can induce antiproliferative effects in HCC cell lines [22]. Knocking out CENP-F inhibits the growth of HCC cells [10]. CENP-H knockout can inhibit the proliferation of Hep3B cells and reduce the colony-forming ability of single cells [23]. In this article, we provide a comprehensive description of CENPs and recent research that has targeted CENPs in HCC, thus providing strategies from a clinical treatment perspective on the basis of CENPs.

## Survey methodology

We accessed Embase, PubMed, and the Web of Science Core Collection for peer-reviewed articles focused on (1) targeted therapy for HCC; (2) the current status of CENP research; and (3) the targeting of CENPs for HCC published from 01/2001 to 06/2024.

1. “Hepatocellular carcinoma” was used as the basic query, and “centromere” or “centromere protein” was added for detailed queries.
2. The “hepatocellular carcinoma” and “targeted therapy” queries were used to search for information about HCC-targeted therapy.

We searched among several original research articles and reviews. Among the 104 publications retrieved from the literature databases for ‘‘hepatocellular carcinoma and centromere’’, 98 articles were classified as basic or clinical trials. The ‘‘hepatocellular carcinoma’’ and ‘‘targeted therapy’’ queries returned 11,211 publications, 151 of which were clinical trials or randomized controlled trials.

In this study, we focused on CENPs in HCC. Therefore, we tended to include the ‘‘centromere protein’’ item. There were 91 articles describing the relationship between CENPs and HCC.

The inclusion criteria were as follows: experimental studies involving animals or humans published in English and listed in literature databases (PubMed, Web of Science Core Collection and Embase) starting from 01 January 2001 and original experimental studies featuring HCC treatment of CENPs.

The exclusion criteria were as follows: publications published before January 1st, 2001, and non-English version articles. Books and documents, summaries, commentaries, editorials, and duplicate studies were also excluded.

The earliest article that included both ‘‘centromere protein’’ and ‘‘hepatocellular carcinoma’’ was published in 2001. From 01/2001 to 06/2024, a total of 91 articles met this requirement. We classified and summarized CENP exposure (structural features, biological effects) and their influence on HCC.

### Structural and functional characteristics of the centromere

**Fig. 1:**
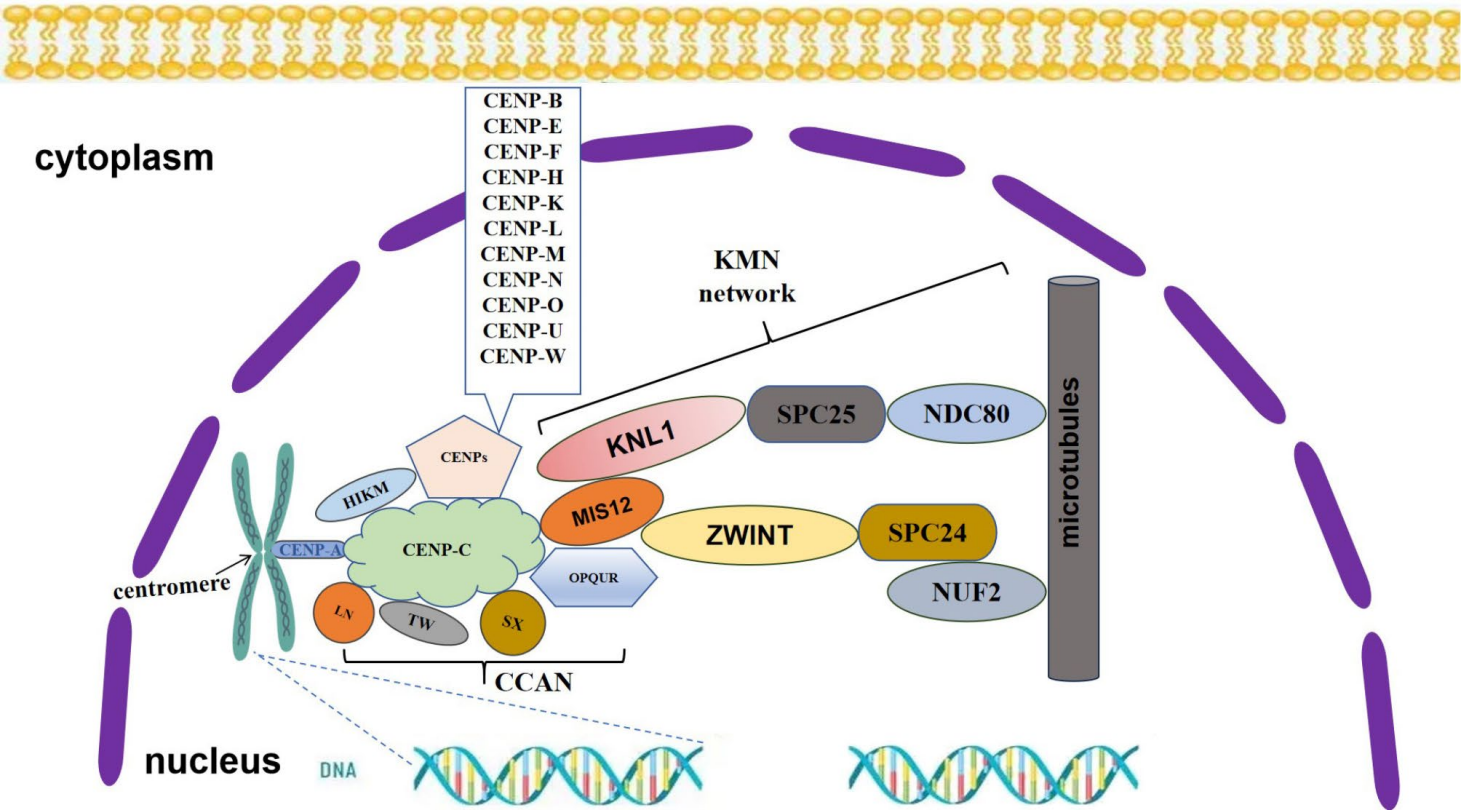
The maintenance and kinetochore tissue of CENP-A in humans. The picture shows the connection between the kinetochore tissue of mitosis and spindle microtubules. The centromere proteins involved in hepatocellular carcinoma are also demonstrated

In the past few years, research on centromeres in various eukaryotes has shown that centromere sizes range from 125 bp to megabases and can be distributed throughout chromosomes [6, 24, 25] (Fig. 1). Centromeres may consist of unique sequences, transposons, or highly repetitive sequences. They can be genetically determined through primary DNA sequences or epigenetically maintained through the localization of nucleosomes containing centromere-specific histone H3 variants (cenH3), which is also called CENP-A, to replace traditional nucleosome histone H3 [8]. CENP-A plays crucial roles in centromere specification, centromere maintenance, and kinetochore assembly in most eukaryotes. Centromeres are typically located in silenced or gene-free chromosomal regions but may contain genes [26, 27]. They are commonly transcribed at low levels to form noncoding ribonucleic acids (RNAs) that interact with kinetochores [28, 29]. The constructive centromere-associated network (CCAN) formed by 16 CENPs is called the inner kinetochore, and the KNL-MISl2-NDC80 (KMN) complex formed by CENPs is called the outer kinetochore [25]. These two important protein complexes are also known as kinetochores. CENP-A and other constitutive centromere-associated network components, including CENP-C and CENP-T, are necessary for recruiting other kinetochore proteins [25, 30]. Most functional centromeres are labeled by the centromere-specific histone H3 variant CENP-A. The modification of CENP-A is dynamic throughout the cell cycle and helps with the localization and function of CENP-A [8]. To date, posttranslational modifications of CENP-A have been identified. These modifications include acetylation, methylation, SUMOylation, and ubiquitination [31–33]. In vertebrates, CENP-A is loaded into the G1 phase during late mitosis [34]. The cell cycle-dependent phosphorylation state of CENP-A is proposed to be critical for timely CENP-A deposition [35, 36]. The ubiquitination of CENP-A is important because of its stability and limitations. Acetylation, phosphorylation, and methylation of specific centromere histones can affect the localization of other CENPs [37, 38].

### Centromere proteins in hepatocellular carcinoma

At present, research on CENPs in HCC is insufficient. It is focused mainly on CENP-A, CENP-B, CENP-E, CENP-F, CENP-H, CENP-K, CENP-L, CENP-M, CENP-U and ZWINT [10, 12, 23, 39–45]. The basic functions of these CENPs are listed in Table 1. Several existing studies demonstrated the relationship between CENPs and HCC. Below, we provide a detailed introduction to the following content.

**Table 1:** Function of centromere proteins

Centromere protein	Function	References
CENP-A	CENP-A can transform centromeres into complexes of DNA and proteins, ensuring that centromeres remain intact during cell division. The heredity of centromeres needs the help of the transportation of CENP-A nucleosomes to retain the epigenetic markers on each sisters’s chromatid, thus ensuring that the human body has almost the same genome.	Li Y, et al.2011
CENP-B	CENP-B mainly binds to the α - satellite DNA sequence of the centromeres and participates in the assembly of the centromeres. CENP-B is involved in regulating some cellular processes, such as gene expression, DNA replication, and DNA repair. In addition, new research suggests that CENP-B may be involved in the development of cancer.	Wang X, et al.2023
CENP-C	The CCAN is a subcomplex of centromeres that binds to centromeres chromatin and provides a platform for centromeres assembly. The CCAN protein CENP-C is the central hub of centromere/kinetochore tissue. CENP-C promotes centromere/kinetochore assembly.	Hara M, et al.2023
CENP-E	CENP-E is only expressed in cells undergoing mitosis and is an essential protein in the kinetochore complex that helps regulate appropriate chromosome segregation and cell division.	Chung V, et al.2012
CENP-F	CENP-F is a cell cycle associated nuclear antigen. When bound to nuclear proteins such as CENP-E, cytoplasmic motor proteins, MAD1, MAD2, Bub1, and BubR1, CENP-F acts as a subunit of protein complexes responsible for kinetochore assembly, microtubule attachment, microtubule dynamics, and spindle checkpoint signals during mitosis.	Dai Y, et al.2013
CENP-H	CENP-H is a constitutive centromere component located at the centromeres throughout the cell cycle. Due to its co localization with CENP-A and CENP-C, CENP-H is considered an internal centromeres protein. CENP-H regulates the growth of cancer cells through the mitochondrial apoptosis pathway.	Lu G, et al.2017
CENP-K	CENP-K is a component of the kinetochore, located on the inner plate of the centromeres, which facilitates the effective assembly of CENP-A with other centromere components. CENP-K promotes cell proliferation, cell migration, and tumorigenicity.	Wang J, et al.2019
CENP-L	CENP-L is involved in the mitotic process of eukaryotic cells and the development of various types of cancer. CENP-L regulates the proliferation, apoptosis, cell cycle, and glycolysis of cancer cells.	Cui Z, et al.2021
CENP-M	CENP-M encodes a dynamic protein that regulates chromosome separation during cell division. CENP-M promotes cancer cell proliferation and metastasis.	Ren H, et al.2021
CENP-N	CENP-N is related to cell cycle, DNA damage and repair. CENP-N promotes cancer cell growth.	Wang Q, et al.2021
CENP-O	CENP-O is crucial for cell cycle checkpoint signaling during spindle formation, chromosome segregation, and mitosis, and its increased expression is associated with poor cancer prognosis. CENP-O is also associated with cancer chemotherapy resistance.	He K, et al.2022
CENP-U	CENP-U is a centromere binding protein that plays an important role in cell mitosis and cell cycle processes, participating in kinetochore assembly, chromosome separation, and mitosis. CENP-U deficiency can lead to chromosomal attachment defects during mitosis.	Liu Y, et al.2022
CENP-W	CENP-W is a member of CCNA involved in mitosis and plays a vital role in ensuring the accurate assembly of sisters chromosomes before division. CENP-W is related to cancer cell proliferation, migration and invasion.	Zhou Y, et al.2021
ZWINT	The human ZW10 interacting kinetochore protein (ZWINT1) and Zeste White 10 (ZW10) are located at the same site on the kinetochore. ZWINT participates in mediating precise chromosome segregation during mitosis. ZWINT is upregulated in various cancers and is associated with poor prognosis.	Lin T, et al.2021

Note. CCAN, constitutive centromere associated network; CENP, centromere protein; HCC, hepatocellular carcinoma;

#### CENP-A

Basic research on CENP-A has revealed that CENP-A is a variant of histone H3. CENP-A, which is located on centromeric DNA, can recruit other CENPs, making it the basis for kinetochore construction [8, 46]. Multiple studies have confirmed that CENP-A is significantly upregulated in HCC compared with adjacent normal tissues [39, 47–49]. High expression of CENP-A is associated with poor prognosis in HCC patients [39]. Mechanistically, CENP-A transcription is activated and synergistically drives the expression of cyclin D1 and neuropilin-2 with YY1. According to the Kaplan‒Meier plot, high levels of CENP-A mRNA are significantly correlated with the survival rate of HCC patients [47]. Knocking out CENP-A inhibits HCC cell proliferation and growth in vitro by blocking the cell cycle at the G1 phase and increasing apoptosis [49]. In contrast, overexpression of CENP-A promoted HCC cell growth and reduced cell apoptosis. Moreover, CENP-A is positively correlated with histological grade, the Ki-67 index, and P53 immunopositivity [49]. For example, with increasing tumor histological grade, CENP-A expression tends to increase. In hepatitis B virus (HBV)-related HCC, hepatitis B virus x (HBx) protein deficiency, especially the COOH terminal deletion of HBx, is a common event in HCC tissues [48]. The expression of CENP-A in HCC tissue is positively correlated with HBx COOH mutations. The HBx mutation can increase the expression of CENP-A, leading to the occurrence of HCC.

#### CENP-B

CENP-B plays a key role in regulating the cell cycle and contributes to the rapid proliferation of HCC cells. As the pathological stage and histological grade progress, the expression of CENP-B increases. Patients with elevated levels of CENP-B mRNA and protein exhibit shorter overall survival and recurrence-free survival [40].

Compared with single therapy, combination therapy with locked nucleic acid (LNA) ASO (hTR) and CENP-B has the greatest synergistic effect on HCC cells [50]. This research suggests that combination therapy with hTR and CENP-B can simultaneously control multiple pathways, providing a promising strategy for HCC treatment. CENP-B mRNA is highly expressed in HCC tissues. Research indicates that CENP-B is closely related to the prognosis of early HCC patients, especially those with AFP levels less than 400 ng/ml, stage I/II disease and a tumor size less than 5 cm [40].

#### CENP-E

As a driving protein, CENP-E can effectively connect centromeres and microtubules, and its expression level and position in cells are strictly controlled [11]. CENP-E is crucial for maintaining chromosome stability because it effectively stabilizes microtubule capture at kinetochore sites [21].

Interestingly, a previous study indicated that both CENP-E mRNA and protein levels are significantly lower in HCC tissues and HepG2 cells than in normal liver cells (LO2), which differs from other members of the centromere family [51]. In addition, the downregulation of CENP-E significantly promoted the proliferation of HCC cells in vitro and in vivo. CENP-E is an independent prognostic factor for advanced HCC patients. Low expression of CENP-E is significantly correlated with adverse clinical and pathological features in patients [41]. Mechanistically, CENP-E inhibits the proliferation of HCC cells by stopping cell cycle progression and accelerating cell apoptosis in the G1‒S phase. Furthermore, a study from China revealed that the CENP-E inhibitor GSK923295 causes a delay in the cell cycle during mitosis, manifested as chromosomal misalignment and aggregation [22]. Taken together, these results shed new light on the ability of CENP-E to be a useful prognostic biomarker and a promising target for anticancer drugs in HCC patients.

#### CENP-F

CENP-F is currently one of the most extensively studied members of the CENP family [52–54]. CENP-F has a molecular weight of approximately 350 kDa. It is a cell cycle-related nuclear protein with the highest expression in the G2 and M phases of the cell cycle and has previously been shown to be highly associated with malignant tumors [55]. Before entering the M phase, the expression of CENP-F peaks and rapidly decreases after mitosis.

Researchers have reported that CENP-F is highly expressed in HCC [10, 56]. Currently, CENP-F is considered a latent serological biomarker for the early diagnosis of HCC [57–59]. A previous study revealed that the area under the curve (AUC) of CENP-F for the early diagnosis of HCC was 0.826. Specifically, 73.6% of alpha fetoprotein (AFP)-negative early HCC cases were positive for CENP-F autoantibodies. Tumor-related autoimmune reactions may be triggered by early HCC [57]. The measurement of CENP-F may supplement AFP and improve the early diagnosis of HCC. Furthermore, the diagnostic value of amino acids 121–220a, the dominant peptide of the CENP-F antigen, in combination with AFP is particularly high, with an AUC of 0.840, a sensitivity of 81.4%, and a specificity of 72.2% [60]. In alcohol-related HCC, two genes (CENP-F and BUB1B) were screened from among the hub genes through least absolute shrinkage and selection operator (LASSO) and Cox regression analyses, and a dual-gene prognostic model, which displayed good performance, was constructed to predict poor prognosis [61]. However, in a multicenter study from China, which included patients with HCC, patients with cirrhosis, patients with chronic hepatitis B virus, and healthy volunteers, CENP-F failed to demonstrate better diagnostic performance, whether alone or in combination [62]. Hence, further research is needed to determine the accurate diagnostic value of CENP-F for HCC.

In addition, researchers have reported that CENP-F upregulation is positively correlated with the serum AFP concentration, tumor stage, venous invasion, and overall survival rate [56, 63]. Another study evaluated the expression of CENP-F in HCC via a series of databases and revealed that CENP-F is closely related to E2F1 and CDK1 in the regulation of the cell cycle, especially during the G2/M transition phase of HCC mitosis [63]. CENP-F may also promote the expression of the cell cycle regulatory proteins c-Myc and Cyclin D1, further activating the corresponding pathways and improving the proliferation and migration ability of HCC cells [64]. Furthermore, CENP-F promotes the migration of HCC cell lines and the progression of epithelial mesenchymal transition (EMT). CENP-F can cooperate with FOXM1 to mediate the expression of the key downstream molecule POLD1, which encodes the DNA polymerase delta catalytic subunit and promotes the incidence and tumorigenicity of HCC [65]. The overexpression of the CENP-F upstream molecule lymphoid-specific helicase (LSH) promotes HCC growth by activating the transcription of CENP-F. Overexpression of LSH and/or CENP-F is correlated with shorter overall survival and higher cumulative recurrence rates of HCC [66]. However, studies have indicated that amplified CENP-F is not significantly related to clinical pathological parameters in HCC, such as age, AFP level, tumor grade, and tumor size, but is more resistant to the chemotherapy drugs 5-FU and doxorubicin than are other drugs [67].

Collectively, these results suggest that CENP-F may be a potential prognostic biomarker and a new therapeutic target for HCC. Nevertheless, further research is needed to validate these discoveries and facilitate the clinical application of CENP-F in HCC.

#### CENP-H

CENP-H was initially identified as the basic component of the active centromere in mouse centromeres [68]. CENP-H may play a key role in the assembly and function of the kinetochore throughout the cell cycle [69]. Immunofluorescence assays revealed that CENP-H is localized in the nucleus of Hep3B cells [20]. Owing to its colocalization with CENP-A and CENP-C, CENP-H is considered an endocentric protein. The CENP-H-I complex is necessary for effectively binding newly synthesized CENP-A to the centromere [70].

Many studies have confirmed that CENP-H is also closely related to the prognosis of patients with HCC [20, 69]. In 60 HCC tissues, the mRNA and protein levels of CENP-H were greater than those in adjacent noncancer samples. High CENP-H levels are related to tumor size, histological grade, late TNM stage and poor prognosis [20]. Mechanistic studies suggest that CENP-H may participate in the proliferation and apoptosis of HCC cells via the mitochondrial apoptotic pathway [23].

#### CENP-K

CENP-K is a member of the CENP-HIK complex [68]. In the absence of CENP-H, the cell cycle stagnates. Researchers have reported that the mRNA and protein levels of CENP-K are significantly elevated in HCC tissue and that their mRNA expression levels are positively correlated with the AFP level (≥ 400 ng/mL) and tumor size (≥ 3 cm). Overexpression of CENP-K stimulates tyrosine phosphorylation of the AKT and MDM2 proteins but inhibits tyrosine phosphorylation of the TP53 protein [42]. These data suggest that the upregulation of CENP-K may be regulated by epigenetic events and contribute to the occurrence of HCC. Additionally, YAP1 is mechanistically responsible for knocking down the tumor suppressive effect of CENP-K in HCC cells. Notably, the inhibitory effects of CENP-K silencing on cell proliferation, invasion, migration and EMT are partially reversed through the recovery of YAP1 expression [71].

#### CENP-L

CENP-L is significantly upregulated in HCC tissue and is associated with various clinical and pathological features and poor patient prognosis [9, 72, 73]. Among males who are not infected with hepatitis virus, the higher the CENP-L mRNA level is, the poorer the overall survival rate. Furthermore, the expression of CENP-L is positively correlated with the level of tumor-infiltrating lymphocytes [73]. Univariate and multivariate analyses suggest that CENP-L may be an independent prognostic factor for HCC [43]. Mechanistic studies have shown that CENP-L functions mainly through the MAPK signaling pathway and activates the MEK1/2-ERK1/2 signaling pathway to facilitate the proliferation and glycolysis of HCC cells. This study clarifies the role of CENP-L in regulating the cell cycle, cell proliferation, apoptosis and glycolysis in HCC.

#### CENP-M

The expression of CENP-M is significantly increased in patients with HCC, and the diagnostic performance of CENP-M has been proven to be excellent, serving as a supplement to AFP in HCC diagnosis [74]. Moreover, CENP-M is associated with immune infiltration levels and poor prognosis in patients with HCC [44].

According to a previous study, miR-214-3p can directly bind to the long noncoding RNA HCG18 and exert antitumor effects on HCC cells. HCG18 can upregulate the expression of CENP-M by acting as a sponge for miR-214-3p [75]. Therefore, these results indicate that HCG18 promotes the proliferation and migration of HCC cells through the miR-214-3p/CENP-M axis. In another study, long nonprotein coding RNA 882 (LINC00882) adsorbs miR-214-3p, thereby promoting the expression of CENP-M [76]. This study identified a new ATF2/LINC00882/miR-214-3p/CENP-M regulatory axis, which may provide potential therapeutic targets for HCC. Furthermore, CENP-M is expressed positively in HCC and is associated with poor prognosis [77]. Mechanistic research has indicated that low expression of CENP-M increases the proportion of cells in the G2/M phase and decreases the proportion of cells in the G0/G1 phase in both the Huh7 and HepG2 cell lines. CENP-M is also associated with the P53 signaling pathway and the cell cycle pathway.

#### CENP-U

The expression of CENP-U in HCC tissues and cells is significantly greater than that in normal tissues and cells [12, 78]. In vitro, CENP-U promotes the proliferation, migration, and invasion of HCC cells [12]. Knocking down CENP-U inhibits the proliferation, metastasis, and G1/S transition of HCC cells both in vivo and in vitro [78]. Mechanistically, CENP-U physically interacts with E2F6 to promote its ubiquitin-mediated degradation, thereby affecting the transcription level of E2F1, further accelerating G1/S conversion and promoting HCC cell proliferation. In addition, the GSEA results indicate that CENP-U is associated with the Notch signaling pathway [12]. KEGG, Reactome, and Wikipathway enrichment analyses revealed that the CENP-U and ZWINT genes are involved mainly in DNA replication and the cell cycle [79].

In HCC, CENP-U and ZWINT are associated with a reduced survival rate [79]. An analysis of HCC datasets from two independent databases revealed that CENP-U can serve as a potential biomarker gene for HCC molecular diagnosis and therapeutic interventions [52, 73].

Taken together, these studies indicate that CENP-U may play a crucial role as a predictive biomarker and therapeutic target for HCC.

#### CENP-W

CENP-W plays a key role in the cell life cycle as a centromere component. It is overexpressed in HCC [80]. Compared with high expression of CENP-W, low expression of CENP-W is associated with a better prognosis in HCC patients. CENP-W inhibition inhibited cell proliferation, migration and invasion. In addition, the percentage of apoptotic cells increases, and liver cancer cells are blocked in the G2/M phase of the cell cycle [80]. However, in another study, knocking down CENP-W suggested that CENP-W can induce G0/G1 phase arrest and cell apoptosis in HCC cells via E2F signaling regulation [81]. Among the prognostic comparative HCC (pcHCC) genes, the upregulation of pcHCC genes is related to prognostic clinical features, consisting of vascular invasion, large tumor size and late HCC stages. Moreover, the pcHCC gene CENP-W, when knocked down, reduces HCC cell viability via disruption of the p38/STAT3 axis combined with sorafenib treatment, thereby hypersensitizing HCC cells [82]. This study provides innovative targets for the development of therapeutic strategies combined with sorafenib on the basis of the different synergistic mechanisms of HCC tumor inhibition.

#### ZWINT

ZWINT is one of the components of the KNLl complex and serves as the essential component of the spindle assembly checkpoint [45, 83]. In recent years, ZWINT mRNA and protein expression were found to be upregulated in HCC samples and some liver cancer cell lines and are significantly correlated with tumor size and quantity [83]. Upregulation of ZWINT is significantly associated with adverse clinical and pathological features, a greater tendency for tumor recurrence and lower survival rates in HCC patients [45, 83]. The results of gene set enrichment analysis indicate that ZWINT and its related genes may be components of condensed chromosomes and spindles, participating in biological processes and signaling pathways such as DNA replication, cytoplasmic division, and the cell cycle checkpoint [45, 79]. ZWINT may be a promising biomarker for poor prognosis and a therapeutic target for HCC. Interestingly, another study revealed that, compared with that in adjacent cancer tissues, the protein expression level of ZWINT in HCC tissue is lower [84]. An analysis of patient prognosis revealed that patients with low expression of ZWINT in HCC have a higher mortality rate and shorter recurrence time. These studies have shown different results, and the expression and significance of ZWINT in HCC require further research.

Four genes (ZWINT, CCNA2, KIF4A and PBK) were effectively identified in the International Cancer Genome Consortium (ICGC) cohort and the Cancer Genome Atlas (TCGA) cohort [85]. These markers can effectively estimate the overall survival rate and aid in prognostic risk assessment. Gene ontology and Kyoto Encyclopedia of Genes and Genomes (KEGG) pathway analysis revealed that ZWINT, CDK2, FEN1, GINS2, GMPS, EZH2, MTHFD1L, MAPKAPK5-AS1 and SRC are significantly upregulated in HCC patients. Analysis of subject operating characteristics revealed that ZWINT, CDK2, MTHFD1L, MAPKAPK5-AS1 and SRC have significant diagnostic value for HCC [86]. Furthermore, a prognostic model involving six genes (ZWINT, CDKN3, DLGAP5, HMMR, KIF20A and NUSAP1) was established, and it may be an independent prognostic factor for HCC patients. The AUC of the model shows that the predictive ability of the model is better than that of other clinical indicators [87].

### Other centromere proteins

CENP-N is also a key gene affecting the occurrence of HCC. Research has shown that a high level of CENP-N expression is an independent risk factor for poor prognosis in patients with HCC [88]. CENP-N expression is associated with a variety of pathways, such as the Rb1 pathway and p53 signaling pathway, in the cell cycle.

CENP-O is upregulated in most cancers, including HCC. The higher the expression of CENP-O is, the worse the prognosis of HCC patients [89]. Mechanistically, CENP-O can activate G2M checkpoints and other signaling pathways. Furthermore, CENP-O expression is associated with HCC immune cell infiltration, immune checkpoint-related molecules and CENP-O promoter methylation. CENP-O may be regarded as a potential biomarker for HCC diagnosis.

### Targeting CENPs for HCC treatment

To date, CENPs have emerged as prospective agents for treating HCC. Drug or small RNA development against CENPs has become a hotspot. Furthermore, some CENPs, such as CENP-B, CENP-E, and CENP-F, have been widely studied, and certain drugs have been evaluated previously in animal experiments and clinical trials (Table 2 and Table 3).

**Table 2:** Examples of interference tools targeting centromere proteins in vitro model in hepatocellular carcinoma

Interference tools	Centromere protein	Cell types	Main results	References
siRNA	CENP-A	HepG2 cells	Knockdown of CENP-A in HepG2 cells reduced cell proliferation, blocked cell cycle at the G1 phase, and increased apoptosis.	Li Y, et al.2011
siRNA (shCENPB#3)	CENP-B	Hep3B and MHCC97 cell lines	shCENPB#3 exhibited a significant inhibition of cell proliferation and invasion capacity in HCC cell lines upon downregulation of CENP-B expression.	Wang X, et al.2023
miR-29a	CENP-B	Hep3B and MHCC97 cell lines	miR-29a may act as a suppressor for HCC by negatively regulating CENP-B expression.	Wang X, et al.2023
GSK923295	CENP-E	LM3, HUH7, and HepG2 cell lines	GSK923295 induced antiproliferation in HCC cell lines. Exposure of liver cells to GSK923295 resulted in delay on a cell cycle in mitosis with a phenotype of misaligned chromosomes and chromosomes clustered.	Tang JC, et al.2019
esiRNA (No. EHU047311)	CENP-F	HCCLM3 and Huh7 cells	CENP-F knockdown significantly inhibited the growth of HCCLM3 and Huh7 cells and inhibited Cyclin E1 and Cyclin B1 expression in HCC cells.	Chen H, et al.2022
double-stranded siRNAs	CENP-F	PLC8024 and SMMC7721 cells	Knockdown of CENP-F could also significantly reduce the abilities of HCC cells to form colonies, as evident in the foci formation assay.	Dai Y, et al.2013
siRNA	CENP-H	Hep3B cells	CENP-H knockdown suppressed the colony formation ability and induced apoptosis of the Hep3B cells.	Lu G, et al.2017
shRNA	CENP-K	BEL-7404 and SMMC-7721 cells	CENP-K downregulation inhibited cell viability and reduced the number of colonies formed by BEL-7404 and SMMC-7721 cells. Silencing CENP-K decreased the migration and invasion of HCC cells.	Wang J, et al.2019
miR-214-3p	CENP-M	Huh-7 and MHCC97-H cell lines	CENP-M mRNA and protein in HCC tissues was significant increased. miR-214-3p could negatively regulate the expression of CENP-M in HCC cells.	Zou Y, et al.2020
miRNA-214-3p	CENP-M	Huh7 and HepG2 cells	miRNA-214-3p distinctly suppressed the expressions of CENP-M, while its silence displayed an opposite result.	Ren H, et al.2021
si-CENPM	CENP-M	Huh7 and HepG2 cells	si-CENPM suppressed the expressions of CENP-M in Huh7 and HepG2 cells. Knockdown of CENP-M distinctly suppressed the proliferation, invasion and migration of Huh7 and HepG2 cells.	Ren H, et al.2021
si-CENPM	CENP-M	HepG2, SMMC-7721, LM3, MHCC-97 H cells	Silencing CENP-M by si-CENP-M significantly suppressed the proliferation, migration and invasion ability of HCC cells.	Duan J, et al.2021
TMEM106C	CENP-M	HepG2, SMMC-7721, LM3, MHCC-97 H cells	TMEM106C significantly suppressed the proliferation and metastasis of HCC through targeting CENP-M.	Duan J, et al.2021
siRNA (shRNA-CENPM)	CENP-M	Huh7 and HepG2 cells	Knocking down CENP-M inhibited cell proliferation, migration and invasion. Low-expression of CENP-M increased the proportion of cells in G2/M phase, and decreased the proportion of cells in G0/G1 both in Huh7 and HepG2 cell lines.	Xiao Y, et al.2019
siRNA	CENP-N	HepG2 and Huh7	The expression of p53, p27, p21, CDK4, cyclin D1, CDK2, cyclin E, pRb, E2F1, and c-Myc decreased after CENP-N knockout. In addition, irradiated CENP-N knockout cells showed a significant increase in γ - H2AX expression and a decrease in colony formation.	Wang Q, et al.2021
siRNAs	CENP-U	Huh-7 and MHCC-97 H cells	Knockdown of CENP-U inhibited the G1/S transition of HCC cells via E2F1.	Liu Y, et al.2022
siRNAs	CENP-W	Hep3B and Huh7 cells	Low expression of CENP-W was associated with better prognosis in HCC patients. SiRNA transfection could effectively knock out CENP-W in liver cancer cells.	Zhou Z, et al.2020
siRNAs	CENP-W	Hep3B and Huh7 cells	CENP-W knockdown could inhibit cell proliferation, migration and invasion by inducing the G0/G1 phase arrest and cell apoptosis in HCC cells via the E2F signaling regulation.	Zhou Y, et al.2021

Note. CENP, centromere protein; siRNA, small interfering RNA; esiRNA, endoribonuclease prepared siRNA; shRNA, short hairpin RNA; HCC, hepatocellular carcinoma;

**Table 3:** Examples of interference tools targeting centromere proteins in vivo model in hepatocellular carcinoma

Interference tools	Centromere protein	Human or animal types	Main results	References
GSK923295	CENP-E	HCC in human*	The CENP-E inhibitor, GSK923295, had a low number of grade 3 or 4 adverse events and the low incidence of myelosuppression and neuropathy.	Chung V, et al.2012
GSK923295	CENP-E	C57BL/6 mice	In mouse model, GSK923295 treatment remarkably reduced liver regeneration in later stage.	Tang JC, et al.2019
shCENPF (Huh7 cells)	CENP-F	NOD-SCID mice	In animals, CENP-F knockdown significantly reduced tumor size and weight.	Chen H, et al.2022
shCENPF-7721 (SMMC7721 cells)	CENP-F	Nude mice	CENP-F knockdown had a significantly retarded ability to initiate tumor formation.	Dai Y, et al.2013
siRNA (LV3-CENP-H)	CENP-H	BALB/c nude mice	In animal study, the average tumor weight and tumor volume in the LV3-CENP-H1 group were lighter and smaller than in the control group. In addition, the LV3-CENP-H1 tumors had a lower Ki-67 expression.	Lu G, et al.2017
shRNA (BEL-7404-shCENPK cells)	CENP-K	The male athymic nude mice	The average tumor volume and weight were lower in the BEL-7404-shCENPK group than in the control group. The tumors formed by BEL-7404-shCENPK cells grew significantly slower than those formed in the control group cells.	Wang J, et al.2019
siRNA (HCCLM3 cells)	CENP-M	male BALB/c nude mice	Knockdown of CENP-M repressed tumor growth and markedly decreased positivity for CENP-M and Ki67, but increased the expression of Bax and C-caspase3.	Xiao Y, et al.2019
sh-CENPU	CENP-U	male BALB/c nude mice	The volume and weight of xenograft tumors decreased visibly following stable silencing of CENP-U.	Liu Y, et al.2022

Note. *clinical trials in human

CENP, centromere protein; siRNA, small interfering RNA; esiRNA, endoribonuclease prepared siRNA; shRNA, short hairpin RNA; HCC, hepatocellular carcinoma;

In a cell-based experiment, transfection of CENP-A small interfering RNA (siRNA) significantly reduced the expression level of endogenous CENP-A in HepG2 cells, thus inhibiting HCC cell proliferation and growth [49]. Similarly, the siRNA shCENPB#3 significantly inhibited the proliferation and invasion capacity of HCC cell lines upon downregulation of CENP-B expression. Furthermore, miR-29a can negatively regulate the expression of CENP-B, indicating that CENP-B may be a promising therapeutic target [40].

Importantly, in a clinical trial on solid tumors, including HCC, the novel CENP-E inhibitor GSK923295 demonstrated a low number of grade 3 or 4 adverse events and a low incidence of myelosuppression and neuropathy [90]. GSK923295 can induce antiproliferation effects by delaying the cell cycle during mitosis in HCC cell lines. In a 70% partial hepatectomy mouse model, GSK923295 significantly reduced liver regeneration [22].

In addition, silencing CENP-F through siRNA can reduce cell proliferation, colony formation, and tumor formation in nude mice and lead to cell cycle arrest during the G2/M checkpoint period by downregulating the cell cycle proteins CDC2 and B1 [56]. In another study, knocking out CENP-F through siRNA inhibited the growth of HCC cells in vitro and in vivo [10]. Silencing CENP-F leads to cell cycle arrest in the G2/M phase and inhibits the expression of Cyclin B1 and Cyclin E1. In an animal study, CENP-F knockdown through shCENPF significantly reduced tumor size and weight. In the present study, transfection of CENP-H siRNA inhibited the proliferation of Hep3B cells and reduced the colony-forming ability of single cells. Together, the average tumor weight and average tumor volume in the LV3-CENP-H1 intervention group were lower than those in the control group in the animal study. In addition, the tumors in the LV3-CENP-H1 intervention group presented lower Ki-67 expression than did the tumors in the control group [23].

The downregulation of CENP-K through shRNA significantly inhibited cell viability and reduced the number of colonies formed by BEL-7404 and SMMC-7721 cells. Silencing CENP-K dramatically decreases the migration and invasion of HCC cells. In male athymic nude mice, the average weight and tumor volume were lower in the BEL-7404-shCENPK group than in the control group. During the entire observation period, the tumors formed by the BEL-7404-shCENPK cells grew significantly slower than those formed by the control cells [71].

According to previous studies, the expression of CENP-M is negatively related to the level of miR-214-3p. miR-214-3p can negatively regulate the expression of CENP-M in HCC cells [75]. Hence, CENP-M is a direct target of miR-214-3p in HCC cells. LINC00882 is significantly upregulated in HCC cells and clinical specimens. Knocking down LINC00882 inhibited the proliferation, invasion, and migration of HCC cells. Mechanistically, LINC00882 adsorbs miR-214-3p, thereby promoting the expression of CENP-M [76]. Additionally, TMEM106C is overexpressed in HCC, and it can significantly inhibit HCC proliferation and metastasis by targeting CENP-M and DLC-1 [91]. In another study, transfection of CENP-M siRNA inhibited cell proliferation, migration and invasion [77]. Together, these findings suggest that CENP-M can serve as a new potential biomarker and therapeutic target for HCC.

Knocking down CENP-N via siRNA reduces the proliferation and colony formation ability of HepG2 and Huh7 cells. Western blot results revealed that the expression of c-Myc, p21, p27, p53, CDK2, CDK4, cyclin D1, cyclin E, pRb, and E2F1 decreased after CENP-N knockout. In addition, irradiated CENP-N knockout cells exhibited a significant increase in γ-H2AX expression and a decrease in colony formation [88].

Knocking down CENP-U via siRNA inhibits the proliferation, metastasis, and G1/S transition of HCC cells both in vivo and in vitro [78]. CENP-W siRNA transfection can also effectively inhibit the proliferation, migration and invasion of liver cancer cells [80, 81]. Taken together, these studies reveal that CENP-U and CENP-W can play critical roles as therapeutic targets for HCC.

## Conclusions

Traditional treatment methods for HCC, including interventional treatment, targeted treatment, immunotherapy, and surgical treatment, have difficulty further improving the survival rate of HCC patients [92]. Thus, new treatment methods are urgently needed. Given that the occurrence of HCC is related to abnormal protein expression, targeted treatment of abnormal protein-related genes is currently a research hotspot.

It has been preliminarily discovered that certain members of the CENP family are abnormally expressed in HCC. Current cellular and animal studies have shown that CENPs can serve as early diagnostic markers for HCC and have good prognostic value. For example, models created with CENP-M or ZWINT as one of the main factors have significant effects on the early diagnosis and prognosis of HCC, with an AUC of up to 0.9 [74, 86, 87]. The model created with CENP-M as the main factor can serve as a supplement to the early diagnosis and prognosis judgment of AFP for HCC. Therefore, we can consider combining different CENPs in clinical practice to improve the diagnostic and prognostic value of HCC and can also combine AFP and abnormal prothrombin for early diagnosis and prognosis assessment. However, research on CENPs as therapeutic targets still lacks clinical trial data. Given that CENPs are widely present in various cells, targeting CENPs for damage to other tissues and organs requires caution. More clinical trials are needed to confirm the treatment effect of CENPs in HCC patients and to further evaluate treatment-related off-target effects.

SEs are physiological and disease factors with strong epigenetic regulatory functions proposed in 2013 [93]. Previous studies have shown that SEs mediate the occurrence of HCC and strongly promote the development of HCC through various signaling pathways [94]. Many studies in this review have shown that CENPs are highly expressed in HCC. Previous studies have confirmed that the Myc gene can regulate the occurrence and development of HCC [95]. CENP-F may promote the expression of the cell cycle regulatory protein c-Myc, further activating the corresponding pathways and improving the proliferation and migration ability of HCC cells [64]. By acting on the bromodomain-containing protein 4 (BRD4) signaling pathway through the small-molecule inhibitor JQ1, SEs can regulate the downregulation of Myc protein and mRNA levels, thereby preventing the proliferation, invasion, and migration of HCC [96]. We speculate that SEs may mediate the regulation of Myc gene expression by CENPs through BRD4 in HCC. Hence, this is a topic of great interest and needs further confirmation via epigenetic research.

As mentioned above, there are various issues and challenges with the treatment of CENPs in HCC, but existing research on CENPs has provided new treatment strategies for clinical practice. This finding offers high confidence for future research on CENPs in HCC. We look forward to making breakthroughs in this area of research in the future and providing hope for more HCC patients.

## Data Availability

No datasets were generated or analysed during the current study.

## Abbreviations

AFP: Alpha fetoprotein
AUC: Area under curve
BRD4: Bromodomain-containing protein 4
CCAN: Constructive centromere associated network
CENPs: Centromere proteins
DNA: Deoxyribonucleic acid
EMT: Epithelial mesenchymal transition
HBV: Hepatitis B virus
HBx: Hepatitis B virus x
HCC: Hepatocellular carcinoma
ICGC: International Cancer Genome Consortium
ICH: Immune Checkpoint Therapy
KEGG: Kyoto Encyclopedia of Genes and Genomes
KMN: KNL-MISl2-NDC80
LINC00882: Long gene non protein coding RNA 882
LNA: Locked nucleic acid
pcHCC: Prognostic comparative HCC
RNA: Ribonucleic acid
siRNA: Small interfering RNA
TCGA: Cancer Genome Atlas
